# The relationship between disease-specific psychosocial stressors and depressive symptoms in Huntington’s disease

**DOI:** 10.1007/s00415-023-11982-x

**Published:** 2023-09-11

**Authors:** Hiba Bilal, Ian H. Harding, Julie C. Stout

**Affiliations:** 1 School of Psychological Sciences, and Turner Institute for Brain and Mental Health, Monash University, 18 Innovation Walk, Clayton, VIC 3800 Australia; 2 Monash Biomedical Imaging, Monash University, Clayton, VIC Australia; 3 Department of Neuroscience, Central Clinical School, Monash University, Prahran, VIC Australia

**Keywords:** Huntington’s disease, Depression, Psychosocial stressors, Psychiatry, Mental health

## Abstract

**Background:**

Huntington’s disease (HD) is an inherited neurodegenerative disease involving motor abnormalities, cognitive decline, and psychological difficulties. Depression is among the most common psychological difficulties in HD. People with HD encounter numerous stressors related to their diagnosis and the impact of HD on their daily lives. Understanding the relationship between HD-specific psychosocial stressors and depression symptoms is critical for optimising treatment and developing a holistic, disease-specific model of depression in HD.

**Methods:**

Fifty-seven adults with the HD gene expansion (33 pre-symptomatic, 24 symptomatic) completed a self-report depression questionnaire and rated how much stress they experienced in relation to 20 psychosocial challenges commonly associated with HD. We examined associations between depression symptoms and each stressor individually, and after clustering using principal components analysis.

**Results:**

Depression symptoms were significantly associated with most of the psychosocial stressors assessed. Clustering with principal components analysis revealed that higher depression scores had significant independent associations with greater stress related to the future implications of HD (β = .44, *p* = .001) and sleep and psychological difficulties (β = .28, *p* = .005), but not with stress related to functional limitations (β = .11, *p* = .33) or interpersonal issues caused by HD (β = .15, *p* = .21).

**Conclusions:**

Stressful experiences associated with HD constitute an important risk factor for depression in HD. Our findings support the use of more psychologically informed models of depression in HD and necessitate further research on tailored psychosocial interventions for HD patients with depression.

**Supplementary Information:**

The online version contains supplementary material available at 10.1007/s00415-023-11982-x.

## Introduction

Huntington’s disease (HD) is a hereditary neurodegenerative disease characterized by progressive motor and cognitive decline, and psychological difficulties [1]. HD is caused by an abnormal expansion in the number of cytosine–adenine–guanine (CAG) triplet repeats in the huntingtin gene [1]. The CAG expansion causes an abnormal form of the huntingtin protein to be coded and expressed throughout the body, causing neuronal death and widespread brain atrophy as HD progresses [2]. HD CAG expansion carriers typically start life with subtle or no disease signs and symptoms, which is referred to clinically as the pre-symptomatic or pre-manifest stage [3]. Clinical signs and symptoms of HD typically emerge when CAG expansion carriers are in their forties, ushering in the symptomatic, or manifest phase [1]. Although treatments are available for managing specific symptoms of HD, no treatment has yet been proven to modify the course of HD itself [2].

Psychological difficulties, such as apathy, irritability, anxiety, and depression, affect most HD CAG expansion carriers at some point in their lifetime [4]. Depression is one of the most common psychological difficulties reported in HD, and affects 33–69% of HD CAG expansion carriers [5]. Cohort studies indicate that the incidence of depression is highest just prior to clinical diagnosis of HD, and in the early stages of HD [6–9]. Depressive symptoms are associated with accelerated functional and cognitive decline, poorer quality of life, self-reported sleep disturbances, and suicidal ideation and behaviour [10–14]. Management of depression in HD is based on treatment guidelines for depression in neurologically healthy populations, and usually involves a combination of pharmacological treatment and psychosocial interventions [15].

The aetiology of depression in HD is complex and appears to involve both biological and psychosocial factors [16]. Yet, a predominantly biomedical framework is used to understand psychological difficulties in HD, wherein syndromes like depression are attributed primarily to the pathophysiological changes that underpin HD progression [8, 17]. As a result, much of the research investigating the correlates of depression in HD has focused on biological factors, such as structural and functional brain changes [18–20], neurotransmitter abnormalities [21, 22], and neuroendocrine dysfunction [23, 24]. Further, many HD CAG expansion carriers attribute their psychological difficulties to biological changes associated with HD, often because of the way these difficulties have been explained to them by their treating clinicians and HD researchers [25, 26]. To some extent, the biomedical view of depression in HD might also contribute to the high prevalence of antidepressant medication use among HD patients with depression [27, 28].

Psychosocial factors associated with depression have received comparatively less attention in HD research. Yet, HD clinicians and patients widely agree that people who inherit the HD CAG expansion experience a range of substantial stressors across their lifetime, such as growing up in a family affected by HD, discovering that one is a HD CAG expansion carrier, experiencing physical, cognitive and behavioural impairments as HD progresses, losing independence and a sense of purpose, and worrying about passing the HD CAG expansion to offspring [29–33]. A large body of literature indicates that exposure to psychosocial stressors, in the form of chronic stress and major adverse life events, can lead to the onset and recurrence of depression [34–36]. Exposure to HD-related ongoing stress and adverse life events might similarly precipitate, maintain, or exacerbate depressive symptoms in HD CAG expansion carriers [25, 37].

In this study, we comprehensively assessed the relationship between stress and depression in HD, with consideration of psychosocial stressors that are unique to those who have inherited the HD CAG expansion. The aims of this study were to investigate whether psychosocial stressors commonly associated with HD are related to depression symptoms in pre-manifest and early manifest HD. We also examined which types of psychosocial stressors have the strongest independent associations with depression symptoms in HD, and whether these associations differ between pre-manifest and early manifest HD groups. In this study, we focused only on recent or current psychosocial stressors (i.e., stressors experienced in the past month) in relation to depression symptoms.

## Methods

### Participants

Fifty-seven people in the pre-manifest and early manifest stages of HD (CAG repeat length ≥ 36) were recruited in Australia through the Experimental Neuropsychology Research Unit and Clinical Cognitive Neuroscience (ENRU-CCN) Laboratory Research Participant Registry, HD specialist clinics, HD advocacy groups, and social media. All participants were Caucasian and native speakers of English. Participants who had not yet met criteria for clinical diagnosis of HD based on motor symptoms were classified as pre-manifest (*n* = 33). Two pre-manifest participants had a CAG repeat length ranging from 36 to 38, which indicates reduced penetrance of the huntingtin gene [38]. On balance, we decided to include these two participants because their experiences of psychosocial stressors in the context of HD were highly relevant to this study. Early manifest participants (*n* = 24) were in Stage I or II of HD (Total Functional Capacity [TFC] Score ≥ 7) [39]. The TFC scale assesses the ability to independently carry out activities such as domestic chores, management of finances, personal grooming, and hygiene. Total scores can range from 0 to 13, with a lower score indicating poorer functional capacity and greater disease severity. For all participants whose genetic data were available, a Disease Burden Score (DBS; Age × (CAG—35.5)) was calculated to estimate lifelong exposure to mutant huntingtin [40]. Exclusion criteria included severe cognitive or communicative impairment which may compromise the ability to complete study measures, psychological disorders other than depression and anxiety, neurological disorders other than HD, brain injury, excessive alcohol or drug use, and concurrent participation in a clinical drug trial.

Thirty-one participants (54.4%) had previously been diagnosed with major depressive disorder by a psychiatrist, psychologist, or other mental health specialist. Thirty-one participants were taking one or more antidepressant medications at the time of participation. Notably, depression symptoms, as assessed by the Quality of Life in Neurological Disorders (Neuro-QoL) Depression scale (see below), did not significantly differ between groups (Table 1). Relative to the pre-manifest group, manifest participants were significantly older, had greater burden of disease, poorer overall functioning, and poorer cognitive performance (COGTEL, see below).

**Table 1:** Descriptive statistics for HD participants (*N* = 57)

	Pre-manifest (*n* = 33)		Manifest (*n* = 24)	
	M (SD)	Range	M (SD)	Range
Age*	44.3 (10.8)	29–65	54.7 (7.9)	43–66
Sex (*n*, Female)	24 (72.7%)	–	13 (54.2%)	–
Years of education	14.4 (2.7)	10–21	13.8 (3.4)	10–26
CAG	41.6 (2.0)	38–45	42.6 (2.0)	37–46
DBS*	270.4 (87.3)	101.5–450.0	383.7 (97.5)	94.5–535.5
TFC*	12.7 (0.8)	9–13	9.2 (2.1)	6–13
COGTEL*	33.1 (10.3)	10.6–51.4	19.1 (7.5)	8.1–32.4
NQ-Depression				
Raw score	15.0 (6.6)	8–33	16.6 (7.6)	8–38
* T*-score	49.5 (6.2)	36.9–64.6	50.5 (7.9)	36.9–69.6

*DBS* Disease Burden Score, *TFC* Total Functional Capacity, *COGTEL* Cognitive Telephone Screening Instrument, *NQ* Neuro-QoL

CAG and DBS data are not reported for three participants (two pre-manifest, one manifest) whose genetic data was unavailable

^*^p < .05 for difference between pre-manifest and manifest groups

The Monash University Human Research Ethics Committee (MUHREC) approved this study (MUHREC ID: 23043). All participants provided written informed consent in accordance with the Declaration of Helsinki [41].

### Measures

#### List of psychosocial stressors in HD

We compiled a list of adverse experiences commonly associated with HD and assessed how stressful participants found each experience in the past month, using an online self-report form (see Table 2). All stressors in the list pertained to current or recent experiences associated with HD. In the list, we included stressors from the Responses to Stress Questionnaire—Huntington’s Disease Version (HD-RSQ) [42]. Other stressors were added to the list based on findings from previous studies assessing which aspects of HD affect quality of life in HD CAG expansion carriers [29–31]. The stressors in the list included specific symptoms of HD (e.g., motor, cognitive, psychological, and sleep-related disturbances), practical and interpersonal difficulties (e.g., financial concerns, the effect of HD on relationships) and negative cognitions related to HD (e.g., concerns about the future/death, losing a sense of meaning or purpose). On a Likert scale ranging from 1 (“Not at all”) to 4 (“Very”), participants rated how stressful each experience had been for them in the past month, with higher ratings indicating higher levels of stress caused by that experience.

**Table 2:** List of psychosocial stressors associated with HD

	Not at all	A little	Somewhat	Very
1. Difficulties with movement	1	2	3	4
2. Difficulty with thinking and communication skills	1	2	3	4
3. Personality changes, aggression, and/or mood swings	1	2	3	4
4. Sleeping difficulties (e.g., not getting enough sleep)	1	2	3	4
5. Not being able to socialise as much with family and/or friends	1	2	3	4
6. Not being able to work or do leisure activities as much as before	1	2	3	4
7. Depending on others as my symptoms get worse	1	2	3	4
8. Concern about what others think of me (now, or as my symptoms get worse)	1	2	3	4
9. Concerns about the future and/or death	1	2	3	4
10. Unstable or chaotic home life due to HD	1	2	3	4
11. Not feeling in control of my life	1	2	3	4
12. Feeling isolated from my friends and/or family	1	2	3	4
13. Grieving the death of a loved one (past or future)	1	2	3	4
14. Not getting enough help or social support	1	2	3	4
15. Passing the HD gene on to my child (or children)	1	2	3	4
16. Losing a sense of meaning or purpose for my life	1	2	3	4
17. Financial concerns	1	2	3	4
18. The effect of HD on my current/future relationships	1	2	3	4
19. Not knowing how, or if, to tell others about my diagnosis	1	2	3	4
20. Worry that my symptoms will get worse over time	1	2	3	4

This is a list of things about having Huntington’s disease (HD) that people sometimes find stressful or a problem to deal with. Please circle the number indicating how stressful the following things have been for you in the past month

#### Quality of life in neurological disorders (Neuro-Qol) depression (short form)

We used the short form of the Neuro-QoL Depression questionnaire [43], which is a self-report measure used to assess depression symptoms in people with neurological disorders, including HD. The Neuro-QoL Depression questionnaire is weighted towards the non-somatic symptoms of depression and is therefore suited to the HD population. Items are rated on a Likert scale ranging from 1 to 5, with higher scores indicating more severe depressive symptoms. Raw scores are converted into standardised *T*-scores with a mean of 50 and a standard deviation of 10, where scores above 50 indicate higher levels of depression. In people with HD, the Neuro-QoL Depression short form has shown excellent internal consistency reliability (Cronbach’s α = 0.96) as well as adequate convergent and discriminant validity [44].

#### Cognitive telephone screening instrument (COGTEL)

The COGTEL [45] is a telephone-based cognitive screening test, which comprises six subtests assessing prospective memory (0–1 point), immediate and delayed verbal memory (0–8 points each), working memory (0–12 points), verbal fluency (unlimited points), and inductive reasoning (0–8 points). Scores from each of the six subtests can be analyzed separately or combined into a weighted total score, with lower scores indicating poorer cognitive functioning. In neurologically healthy older adults, the COGTEL has shown adequate test–retest reliability (*r* = 0.85) and correlates strongly with scores on the Mini-Mental State Examination (*r* = 0.93), demonstrating strong convergent validity [46]. Further, the COGTEL is not limited by ceiling effects [46], and is, therefore, suitable for assessing cognitive functioning in pre-manifest HD participants who have mild or no cognitive impairments.

### Procedure & data analysis

As part of a screening telephone call made by the first author (HB), potential participants were asked a series of questions based on a demographic questionnaire and items from the TFC scale, from which the first author assigned a TFC score. Subsequently, participants who were eligible for the study completed the informed consent process and the remaining study measures electronically via a web-based link to Research Electronic Data Capture (REDCap) [47]. Approximately one week after participants had completed the consent form and other study measures, they completed the COGTEL with HB via telephone.

We performed all statistical analyses using SPSS Version 26. We used Spearman’s rank order correlations to examine associations between depression scores and each of the 20 psychosocial stressors; a non-parametric correlation test was used because of the ordinal nature of the psychosocial stressor ratings. Next, we conducted a principal components analysis (PCA) with Varimax rotation to group together similar psychosocial stressors, thereby minimising item redundancy. The Kaiser–Meyer–Olkin (KMO) measure verified sampling adequacy for the analysis, KMO = 0.81. All KMO values for individual items were above 0.61 (> 0.5, the acceptable limit; [48]). Bartlett’s test of sphericity was significant (*p* < 0.001), confirming the suitability of PCA for the list of stressors.

We performed Mann–Whitney *U*-tests to assess whether mean scores for each stressor and PCA component differed significantly between pre-manifest and manifest groups. We applied Bonferroni adjustments to correct for multiple comparisons in correlational and between-groups analyses. The significance threshold following Bonferroni adjustments was 0.0025 (0.05/20 comparisons) for correlations and between-group differences in stressor ratings, and 0.01 (0.05/4 comparisons) for between-group differences in PCA component scores.

We performed multiple regression analysis to examine the independent associations of each component derived from the PCA with depression symptoms. Interaction effects between each PCA component and disease stage (pre-manifest and manifest) were assessed in separate multiple regression analyses, with the other PCA components included as covariates. Simple slopes analyses were performed to further examine statistically significant interactions. One multivariate outlier was excluded from the multiple regression testing the associations of the four PCA components with depression scores (*N* = 56). Two multivariate outliers were excluded from the regression analyses testing the interaction between PCA components and disease stage for depression scores (*N* = 55).

## Results

### Psychosocial stressor ratings in pre-manifest and manifest groups, and correlations with depression scores

**Fig. 1:**
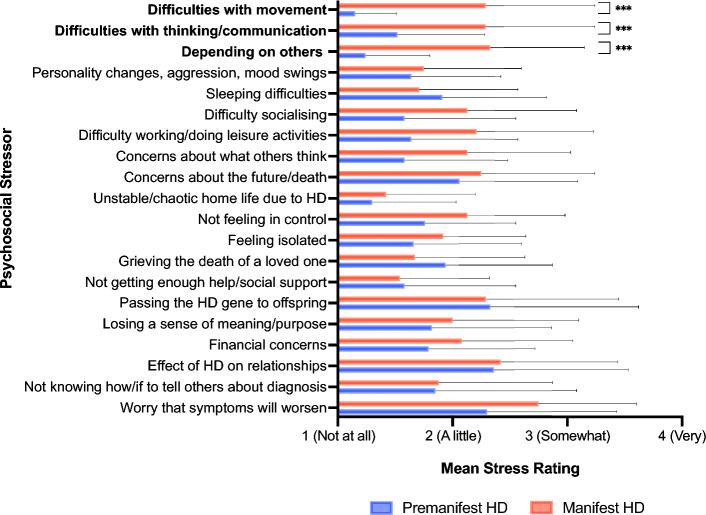
Mean psychosocial stressor ratings in pre-manifest and manifest groups. ***p < .0025 (significance threshold following Bonferroni correction for 20 comparisons). Error bars = standard deviation

As shown in Fig. 1, the manifest group experienced significantly more stress than the pre-manifest group regarding difficulties with movement, difficulties with thinking and communication, and depending on others as HD symptoms worsen. Mean ratings for the remaining stressors did not significantly differ between pre-manifest and manifest groups (*p* > 0.0025).

As shown in Table 3, the majority of stressors assessed in this study (15 out of 20) significantly correlated with higher (more severe) depression scores (*p* < 0.0025), and medium to large effect sizes were observed for all correlations. Depression scores did not significantly correlate with stress ratings for personality changes/aggression/mood swings, sleeping difficulties, dependence on others, passing the HD gene to offspring, or not knowing how/if to tell others about one’s diagnosis (*p* > 0.0025). Supplementary Fig. 1 provides scatterplots depicting the relationship between each stressor and depression scores.

**Table 3:** Spearman’s correlations between Neuro-QoL depression scores and ratings for psychosocial stressors associated with HD

	Spearman’s rho	*p*
1. Difficulties with movement	.45	< .001***
2. Difficulty with thinking and communication	.47	< .001***
3. Personality changes, aggression, and/or mood swings	.39	.003
4. Sleeping difficulties	.36	.006
5. Not being able to socialise as much with family/friends	.55	< .001***
6. Not being able to work or do leisure activities as much as before	.49	< .001***
7. Depending on others as symptoms worsen	.32	.02
8. Concerns about what others think (now or as symptoms worsen)	.53	< .001***
9. Concerns about the future and/or death	.63	< .001***
10. Unstable or chaotic home life due to HD	.41	< .001***
11. Not feeling in control of one’s life	.60	< .001***
12. Feeling isolated from friends and/or family	.48	< .001***
13. Grieving the death of a loved one (past/future)	.49	< .001***
14. Not getting enough help or social support	.56	< .001***
15. Passing the HD gene on to one’s offspring	.32	.02
16. Losing a sense of meaning or purpose for one’s life	.68	< .001***
17. Financial concerns	.61	< .001***
18. The effect of HD on current/future relationships	.62	< .001***
19. Not knowing how/if to tell others about one’s diagnosis	.37	.004
20. Worry that symptoms will get worse over time	.53	< .001***

^***^p < .0025 (significance threshold following Bonferroni correction for 20 comparisons)

### Principal components analysis

The principal components analysis indicated four components that had eigenvalues over Kaiser’s criterion of one [48] and in combination explained 68.61% of the variance. Table 4 shows loadings for the four components after Varimax rotation (only loadings above 0.4 were represented). We labelled each component based on the resultant pattern of clustering: Component 1 represents functional limitations caused by HD, Component 2 represents the future implications of having the CAG expansion for HD, Component 3 represents interpersonal issues related to HD, and Component 4 represents sleep and psychological difficulties in HD. Stressors loading onto multiple components were generally assigned to the cluster with which they had a higher loading.

**Table 4:** Loadings of the 20 psychosocial stressors associated with HD onto 4 principal components

	Component			
	1	2	3	4
(1) Functional limitations caused by HD				
Cognitive/communicative difficulties	.84			
Movement difficulties	.78			
Depending on others as symptoms worsen	.78			
Not being able to work/do leisure activities as much as before	.74			
Not being able to socialise as much with family/friends	.73			
Financial concerns	.63		.41	
(2) Future implications of HD				
Concerns about the future/death		.77		
The effect of HD on current/future relationships		.77		
Worry that symptoms will worsen over time		.75		
Passing the HD gene to offspring		.74		
Not feeling in control of my life	.45	.63		
Not knowing how/if to tell others about my diagnosis		.60		
Concern about what others think of me (now/as symptoms get worse)	.56	.59		
Losing a sense of meaning/purpose for my life	.42	.58		
(3) Interpersonal issues related to HD				
Feeling isolated from friends/family	.61		.47	
Not getting enough help/social support			.85	
Grieving the death of a loved one (past/future)		.42	.75	
Unstable/chaotic home life due to HD			.51	
(4) Sleep and psychological difficulties in HD				
Sleeping difficulties				.88
Personality changes/aggression/mood swings				.82

**Fig. 2:**
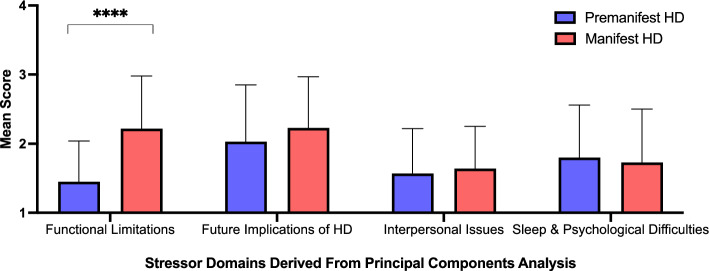
Mean scores for stressor domains derived from principal components analysis. ****p < .001 for between-group differences in component scores

For stress related to Functional Limitations Caused by HD (Component 1), the manifest group had higher scores than the pre-manifest group, *U* = 641.00, *z* = 3.99, *p* < 0.001 (Fig. 2). Pre-manifest and manifest groups did not significantly differ on mean scores for stress related to the Future Implications of HD (*p* = 0.19), Interpersonal Issues Related to HD (*p* = 0.58) and Sleep and Psychological Difficulties in HD (*p* = 0.80).

### Multiple regression analyses with PCA-derived components and depression scores

Multiple regression analysis indicated that the four components derived from the PCA accounted for 62% of the variance in depression scores, *F*(4, 55) = 20.82, *p* < 0.001. As shown in Table 5, higher levels of stress related to the Future Implications of HD (Component 2) and Sleep and Psychological Difficulties in HD (Component 4) had significant independent associations with higher (more severe) depression scores. Moreover, stress related to Future Implications of HD had the strongest independent association with depression scores.

**Table 5:** Regression coefficients of PCA-derived components on depression scores in HD (*N* = 56)

Component	β	*t*	*p*
1. Functional limitations in HD	.11	0.98	.33
2. Future implications of HD	.44	3.50	.001**
3. Interpersonal issues in HD	.15	1.27	.21
4. Sleep and psychological difficulties in HD	.28	2.93	.005**

^**^p < .01

**Fig. 3:**
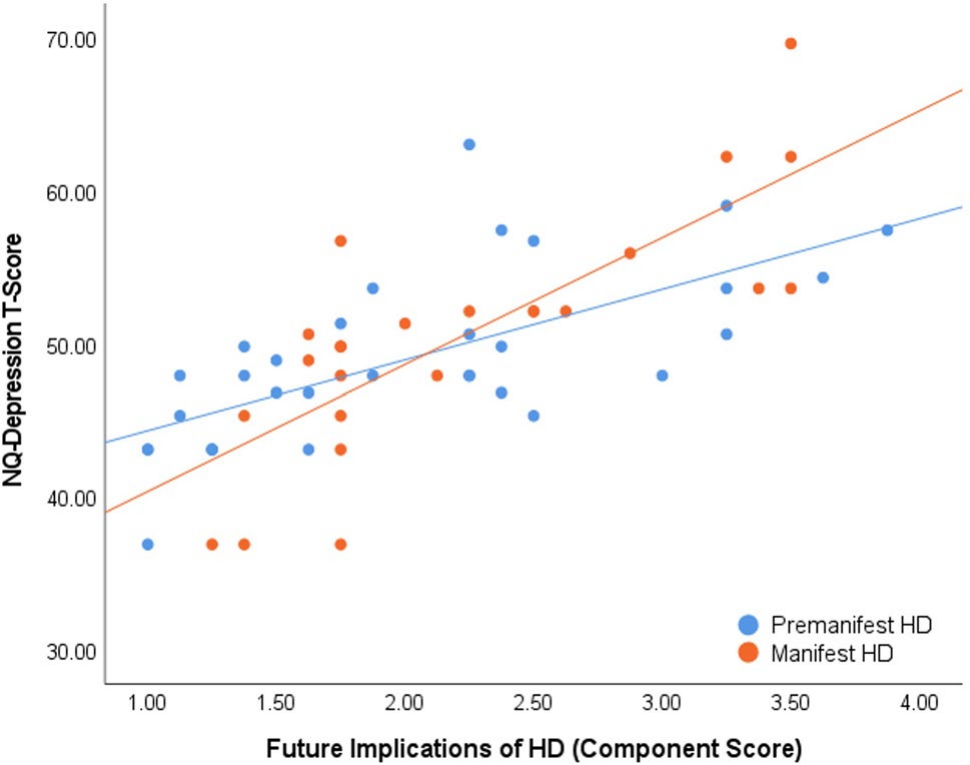
Association between stress related to future implications of HD and depression scores in pre-manifest and manifest HD groups

We observed a significant interaction between stress related to the Future Implications of HD (Component 2) and disease stage for depression scores (β = 0.30, *p* = 0.02). In the manifest group, the independent association between stress related to the Future Implications of HD and depression scores (β = 0.67, *p* = 0.006) was stronger compared to the pre-manifest group (β = 0.35, *p* = 0.05; see Fig. 3). The independent associations between depression scores and stress related to Functional Limitations of HD (Component 1), Interpersonal Issues Related to HD (Component 3) and Sleep and Psychological Difficulties in HD (Component 4) did not differ according to disease stage (*p* = 0.15, *p* = 0.19, and *p* = 0.57, respectively).

## Discussion

This study indicates that depression symptoms are associated with stress regarding numerous psychosocial aspects of HD. Our findings extend previous research showing that perceived stress is associated with depression symptoms in pre-manifest HD [37], and research indicating that many HD CAG expansion carriers attribute their depression to stressors associated with HD [25]. Research in neurologically healthy groups has similarly shown that repeated exposure to stressful events can lead to the onset, maintenance, and recurrence of depression [34, 36], particularly in people who already have a biological predisposition to depression [49, 50]. Consistent with prior literature, the wide range of stressful experiences associated with HD may have a cumulative impact on psychological functioning and eventually precipitate depressive symptoms in HD patients, who may already be more susceptible to depression because of the HD CAG expansion [51, 52].

Currently, a predominantly biomedical framework is used to understand and explain psychological difficulties in HD, despite the contributions of psychosocial factors to these difficulties. Our findings highlight the strong associations of many psychosocial stressors with depression symptoms in HD, suggesting the need for more psychologically informed models of depression in HD. Considering the contributions of psychosocial factors to depression is essential when assessing HD patients, and when providing psychoeducation regarding depression to HD patients, their caregivers, and other members of the HD community. Moreover, adapting psychosocial interventions according to the unique needs and experiences of HD patients is also likely to be beneficial in ameliorating depressive symptoms. Given the scarcity of research on psychosocial interventions for HD [53], the efficacy of different psychological therapies for HD remains unclear. Our findings suggest that psychological interventions tailored to the HD patient’s specific experiences and traumas (e.g., cognitive-behavioural, acceptance and commitment, family, and psychodynamic therapies) may improve depressive symptoms, particularly when administered alongside pharmacological treatments. Further, as recommended by Zarotti, et al. [54], increasing specialist knowledge and education on HD for clinicians, and enhancing HD care staff’s psychological knowledge and skills, can also enable healthcare professionals to respond to HD patients with depression in a more psychologically informed manner.

We report that stress regarding the future implications of HD had the strongest association with depression symptoms, independent of the other psychosocial factors we assessed. Concern regarding the future has emerged as a prominent theme in the narratives of many HD CAG expansion carriers, including concerns about end-of-life issues, passing the HD CAG expansion to offspring, and a future with significant HD symptoms [25, 29, 30]. The wide range of implications associated with a HD diagnosis, coupled with difficulty in predicting what a future with HD will entail, may result in ongoing stress and depressive symptoms in susceptible HD CAG expansion carriers. Therefore, cognitions and emotions regarding the future implications of HD may be a particularly important target for psychological interventions with HD CAG expansion carriers experiencing depression.

Depression symptoms were also associated with stress regarding sleep and psychological difficulties in HD. These findings are consistent with previous research showing that depression symptoms are associated with subjective sleep complaints in HD [13, 55–57], and that emotional issues and sleep disturbances are among the most impactful disease symptoms for HD CAG expansion carriers [29–31]. Given that sleep disturbances and psychological difficulties like aggression, mood swings, and personality changes are largely internal processes, HD CAG expansion carriers may be more aware of and distressed by these issues, compared to more external deficits like motor and cognitive impairments. These findings reinforce the significance of sleep and psychological difficulties for quality of life in HD and highlight the importance of managing these symptoms alongside motor and cognitive deficits.

We also found that the relationship between depression symptoms and stress regarding future implications of HD was stronger in participants with manifest HD, compared to pre-manifest HD participants. In the manifest stage, HD signs and symptoms become more prominent and debilitating, and their implications for one’s future are likely to become more imminent. As a result, impending issues related to HD may begin to bear greater weight and have a stronger psychological impact compared to the pre-manifest stage. Interestingly, the association between depression and other stressor domains did not differ between pre-manifest and manifest groups. A recent study revealed that pre-manifest HD patients report experiencing many of the symptoms that are present in manifest HD, even though they do not yet meet clinical criteria for HD diagnosis [31]. These findings suggest that pre-manifest and manifest HD patients have a similar subjective experience of HD symptoms, which may lead to similar associations between most HD-related stressors and depression in these groups.

In terms of study limitations, we examined only a limited number of psychosocial stressors in relation to depression symptoms, and we measured the impact of each stressor in the past month only. In addition to the psychosocial stressors assessed in this study, depression symptoms may have also been influenced by psychosocial stressors which occurred earlier in life (e.g., childhood trauma resulting from early exposure to HD), or constitute a less common symptom of HD (e.g., pain, fatigue). Stigma is another psychosocial factor that is associated with depression and disease-related outcomes in HD [58], and is also known to mediate the relationship between depression and psychosocial factors in Parkinson’s disease [59]. Although stigma was not assessed comprehensively in this study, it may have influenced the disclosure or reporting of depression symptoms in our sample, as well as the relationships observed between depression and psychosocial stressors in this study. Another limitation of this study was the correlational nature of our findings, due to which the directionality of the stress–depression relationship in this study cannot be established with certainty. For example, participants with more severe depressive symptoms may have been more attuned to the challenges associated with HD than less depressed participants, thereby rating these challenges as more stressful. Further, this study only included participants in pre-manifest and early manifest stages of HD, thus our findings cannot be generalised to people with advanced HD.

In conclusion, this study provides an initial understanding of the relationship between psychosocial stressors unique to HD and depression symptoms in pre-manifest and early manifest HD. Depression symptoms were significantly associated with stress regarding the future implications of HD, as well as stress regarding sleep and psychological difficulties caused by HD. Moreover, the relationship between depression symptoms and stress regarding the future implications of HD was stronger in manifest HD compared to pre-manifest HD. To improve knowledge of the stress–depression relationship within the context of HD, future research should examine a more comprehensive list of stressors in relation to depression symptoms, in a larger and more diverse sample of HD CAG expansion carriers. Future research should also investigate additional moderators of the stress–depression relationship in HD, such as genetic and pathophysiological factors, personality traits, and sociodemographic variables.

### Supplementary Information

Below is the link to the electronic supplementary material.

Supplementary file1 (PDF 764 KB)
