# Supplementary material for: The relationship between disease-specific psychosocial stressors and depressive symptoms in Huntington’s disease
Source: J Neurol. 2023 Sep 11;271(1):289–99. doi: 10.1007/s00415-023-11982-x (PMC10769991; doi:10.1007/s00415-023-11982-x)
Supplement: Supplementary file 1 — Supplementary file1 (PDF 764 KB) [file 415_2023_11982_MOESM1_ESM.pdf]

Supplementary Figure 1

Scatterplots for Associations Between Each Psychosocial Stressor and Depression Severity

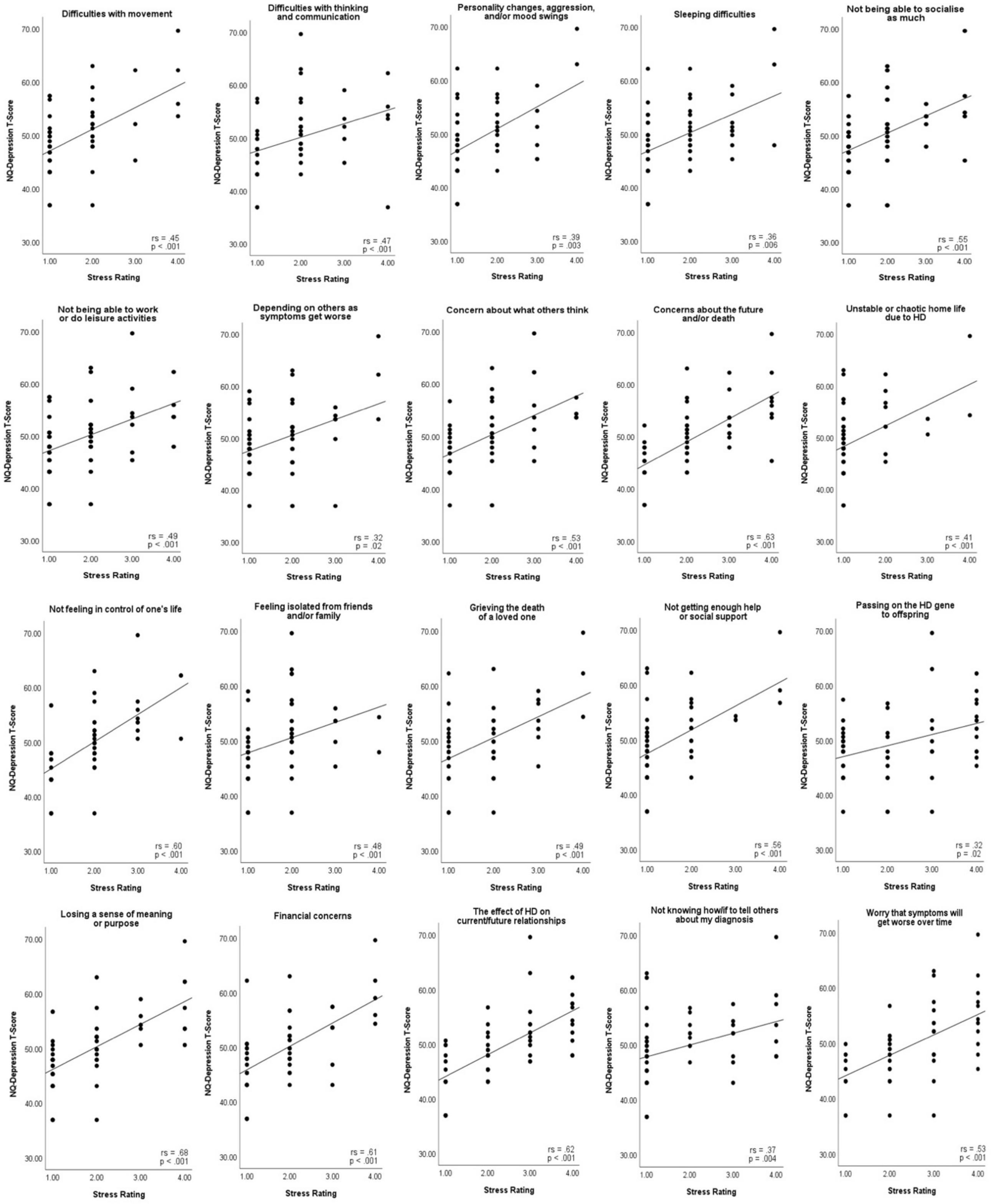

Note. rs = Spearman's rho.
Significance threshold following Bonferroni correction for 20 comparisons = $p < .0025$.

Article Title: The Relationship Between Disease-Specific Psychosocial Stressors and Depressive Symptoms in Huntington's Disease

Journal Name: Journal of Neurology

Author Names: Hiba Bilal, Ian H. Harding, Julie C. Stout

Corresponding Author: Professor Julie Stout, Turner Institute for Brain and Mental Health, 18 Innovation Walk, Monash University, Clayton, 3800, Victoria, Australia. Tel: +61 3 9905 3987; Email: julie.stout@monash.edu
